# Effects of Playback Speed on Eye Movement Behavior During News Viewing: An Eye-Tracking Study

**DOI:** 10.3390/jemr19050101

**Published:** 2026-09-10

**Authors:** Melissa Gurdián-Franceschi, Miguel Ángel Martín-Pascual, Celia Andreu-Sánchez

**Affiliations:** 1 Facultad de Comunicación Social, Universidad de Panamá, Panama City 3366, Panama; 2 Neuro-Com Research Group, Departament de Comunicació Audiovisual i Publicitat, Universitat Autònoma de Barcelona, 08193 Cerdanyola del Vallès, Barcelona, Spain; miguelangel.martin@rtve.es; 3 Research and Development, Instituto Radio Televisión Española, 08174 Sant Cugat del Vallès, Barcelona, Spain

**Keywords:** eye-tracking, news clips, TV news, playback speed, visual perception, cognitive processing, attention, spontaneous eyeblink

## Abstract

The increasing use of accelerated playback in digital media consumption has raised questions regarding its effects on viewers’ perception. This study examined whether playback speed in TV news videos (1× vs. 2×) affects viewers’ subjective responses, memory performance, and eye-tracking behavior. We presented news videos under either normal or accelerated playback conditions to a total of *N* = 146 participants. Our results showed that accelerated playback was associated with lower valence, reduced perceived comprehension, lower credibility evaluations, poorer memory performance, and lower recommendation-intention ratings. In contrast, arousal ratings were higher under accelerated playback conditions. Eye-tracking measures revealed differences in visual attention patterns between conditions, including fixation behavior and attention allocated to lower-third captions. However, the fixation-rate difference should be interpreted cautiously, as increased stimulus velocity may have influenced fixation classification. These findings suggest that accelerated playback was associated with differences in audience evaluation and viewing behavior during news consumption.

## 1. Introduction

In the current technological landscape, digital video services have reshaped how audiences access and engage with audiovisual content. Viewers can now watch videos at any time through a wide range of streaming platforms and smart devices [1,2]. In this context, accelerated playback—particularly at speeds such as 2×—has become increasingly common [3,4]. This practice is often associated with efforts to manage time more efficiently, limit distractions, and maintain greater control over media consumption [5]. Advances in video playback technology have enabled accelerated playback, allowing viewers to process larger volumes of audiovisual material in shorter periods without substantially altering the overall viewing experience [2,6,7,8].

As accelerated playback becomes increasingly common, it is important to examine how this viewing practice affects the processing of audiovisual information. Existing research suggests that viewers can generally understand audiovisual material when it is presented at faster playback speeds, such as 1.25× or 2× [9,10]. Studies focusing on multimedia learning further indicate that comprehension and retention can remain stable at speeds of up to 2×, particularly when content is clearly structured and supported by elements such as subtitles or visual guidance [9,11,12,13,14]. Some findings even suggest that higher playback speeds may improve retention on subsequent assessments [6,7,15]. However, these findings remain subject to debate. While recall performance may improve under certain conditions, memory should be distinguished from comprehension, as the ability to remember information does not necessarily indicate a full understanding of its meaning [16]. Evidence from educational settings also indicates that accelerated playback may be associated with improved academic performance, reduced viewing time, and higher completion rates [15]. Similarly, studies involving medical students have reported no significant differences in concentration when lectures are viewed at faster playback speeds, suggesting that accelerated playback does not necessarily compromise memory performance [10]. Although the effects of faster playback appear to depend on factors such as content characteristics and viewers’ familiarity with this viewing practice, available evidence suggests that increasing playback speed does not necessarily reduce perceived credibility or memorability under all conditions [9,17,18]. Likewise, minor distractions appear to have little effect on performance at playback speeds of up to 2× [9]. Demographic characteristics may also moderate these effects, as younger viewers tend to maintain attention and comprehension at 2× more effectively than older adults [2,6,11,19].

The role of presentation speed has also been examined in relation to subtitle processing within audiovisual content. In multimodal reading contexts, rereading allows viewers to correct potential comprehension errors and integrate verbal information with concurrent visual elements. Accordingly, it has been observed that as subtitle speed increases, viewer comprehension declines. Higher speeds reduce the likelihood of rereading and lead to more incomplete subtitle processing, thereby limiting opportunities for information integration and cognitive processing [20]. Understanding the mechanisms underlying these effects requires examining how the pacing of audiovisual material influences attentional processes. Stimuli characterized by high velocity and visual complexity have been shown to broaden attentional scope while simultaneously reducing conscious processing [21]. In this context, gaze behavior during video viewing reflects both viewer states and characteristics of the audiovisual content. A modeling approach based on eye-tracking has been proposed to integrate video dynamics, gaze dynamics, and viewer states through structural and statistical relationships, enabling the estimation of how viewers process complex audiovisual content [22].

In parallel, the speed of audiovisual presentation can be manipulated through time-compression techniques, which allow changes in the rate of information flow without altering other characteristics of the message. Adjusting the pace of presentation is therefore relevant because some segments may be too slow to maintain viewer interest, whereas others may be too fast to be effectively assimilated. Optimizing the rate of information flow may thus be an important factor in audiovisual communication effectiveness [23]. These considerations are particularly relevant to news production and consumption, where an increasing emphasis on immediacy has contributed to the rise of speed-driven journalism [24]. Within this context, journalists tend to perceive speed as detrimental to credibility and beneficial for news use; however, experimental evidence has not supported these assumptions, as no significant effects have been observed on credibility or future news use. Nevertheless, certain fast-paced news formats, such as live blogs, have been perceived as more difficult to follow, suggesting that the speed at which news is presented may have implications for how audiences process information [24]. This relationship between speed and processing is also reflected in perceptual responses. Faster playback speeds have been shown to reduce perceived urgency in younger audiences, whereas no significant effects have been observed in older viewers or in behavioral intentions [25].

From a cognitive perspective, meaning construction in audiovisual content is not solely determined by visually salient elements. Viewers integrate bottom-up and top-down processes during visual processing [26,27], with attentional allocation influenced by both perceptual salience and narrative relevance. In this context, visually peripheral elements may acquire narrative importance when competing with components of high visual and narrative salience, highlighting the complexity of attentional allocation in dynamic audiovisual environments [28]. Evidence from oculographic studies further indicates that rapid cuts and accelerated video sequences can increase attentional engagement, partly due to reductions in blink frequency [21,29]. At the same time, attentional allocation is shaped by narrative structure, audiovisual synchrony, and editing strategies, which influence perception, emotional engagement, and narrative coherence [30,31,32,33,34,35,36]. In this framework, eye blinks do not occur randomly during video viewing but follow temporal patterns linked to narrative dynamics. Spontaneous blinks have been shown to synchronize across viewers and tend to occur during moments of lower attentional demand, such as the conclusion of an action or the absence of the main character. This suggests the presence of a shared mechanism that regulates blink timing in order to minimize the loss of critical visual information [37]. Blink rate has also been linked to cognitive load and attentional demand, although the direction of this relationship appears to depend on task characteristics [38,39,40]. In visually demanding tasks, reduced blink frequency has been associated with increased mental workload and visual attentional demands [38,39], whereas higher blink rates have been observed under conditions of lower visual demand [38]. Conversely, in a non-visual auditory oddball paradigm, increased cognitive load has been connected to an increase in blink rate [40]. Moreover, although electrophysiological measures of attention also vary across task conditions, these changes do not necessarily correlate with variations in blink rate, suggesting that different physiological markers may capture distinct aspects of cognitive activity [40]. From a methodological perspective, investigating the effects of playback speed therefore requires measures that can complement self-reported responses. Self-reports may not fully capture users’ internal psychological states because of factors such as social desirability bias or limited emotional awareness. Eye-tracking can provide complementary information by capturing eye movement patterns associated with visual processing and attentional allocation during audiovisual viewing [41,42,43].

The present study investigates how accelerated playback speed (2×) influences the perception and processing of news video clips. Although previous research has examined the effects of playback speed on comprehension, memory, attention, and perceptual responses, limited evidence exists on how accelerated playback affects eye movement behavior during news viewing. To address this gap, the same set of informative news videos was presented at two playback speeds—normal speed (1×) and accelerated speed (2×)—to two independent groups of participants. Eye-tracking measures were recorded to examine visual attention during viewing, while subjective responses and memory performance were collected through questionnaires administered during the experimental session. Specifically, the study combined self-reported measures, memory performance, and eye-tracking data to provide complementary information about viewers’ responses to playback speed. The goal of this study was to examine whether TV news playback speed (1× vs. 2×) influences viewers’ subjective evaluations, memory for the information presented, and eye movement behavior during viewing. We hypothesized that these measures would differ between the 1× and 2× playback-speed conditions, but we did not make specific directional predictions regarding the nature of these differences.

## 2. Materials and Methods

### 2.1. Subjects

A total of 172 subjects aged 18–63 years were initially recruited and assigned to one of two experimental conditions: normal-speed playback (1×) or accelerated-speed playback (2×). Playback-speed condition was assigned at the experimental-day level, such that each experimental day was assigned to a single playback-speed condition, and all participants tested on that day received the same playback-speed condition. The allocation procedure was organized to promote comparable group sizes across conditions over the course of data collection. Data collection comprised 55 experimental days, with 32 days assigned to the 1× condition and 23 days assigned to the 2× condition. Of these, 44 experimental days were conducted at the Neuro-Com Research Group laboratory at the Universitat Autònoma de Barcelona (27 days under 1× and 17 days under 2×), and 11 days were conducted at the facilities of Radio Televisión Española in Barcelona, Spain (5 days under 1× and 6 days under 2×). Following data–quality screening, 26 individuals were excluded because of inadequate eye-tracking signal quality, unsuccessful calibration, or voluntary withdrawal from the experimental session (see Section 2.7, Data Collection and Pre-Processing, for more details). Of these, 21 participants were excluded from the 1× condition and 5 from the 2× condition. Because all participants tested on a given experimental day were assigned to the same playback-speed condition, this unequal attrition may partly reflect day-specific variations in eye-tracking signal quality. The final sample comprised 146 participants (*N* = 146), with 66 assigned to the normal-speed condition (1×; *n* = 66) and 80 assigned to the accelerated-speed condition (2×; *n* = 80) (see Table 1). Participants in the 1× condition had a mean age of 30.74 years (SD = 12.42), whereas those in the 2× condition had a mean age of 37.35 years (SD = 14.21). In the 1× condition, 41 participants were female, 24 were male, and 1 identified as other; in the 2× condition, 53 were female, and 27 were male. Altogether, the final sample included 94 women (64.4%), 51 men (34.9%), and 1 participant identifying as other (0.7%), with no missing data. Mean age was 33.84 years (*SD* = 13.62) for women and 35.65 years (*SD* = 14.09) for men. Recruitment was conducted through email announcements, posters displayed on campus, and direct invitations in hallways and classrooms. Participation was voluntary and unpaid, and appointments were arranged through an online scheduling system.

Given the final sample sizes of 66 participants in the 1× condition and 80 participants in the 2× condition, a sensitivity analysis was conducted using G*Power, version 3.1 (Heinrich-Heine-Universität Düsseldorf, Düsseldorf, Germany) to determine the minimum detectable effect size for a two-tailed independent-samples *t*-test, assuming α = 0.05 and a statistical power of 0.80. The analysis indicated that the study could detect effects of approximately Cohen’s *d* = 0.47 or larger, corresponding to effects approaching the conventional medium range [44].

### 2.2. Ethical Approval

The study complied with the institutional ethical regulations governing research involving human participants at the Universitat Autònoma de Barcelona (UAB). Ethical approval for the research protocol was granted by the Ethics Commission for Research with Animals and Humans (CEEAH/CERec; file number 6370). Data collection began on 21 March 2025. The approval remained valid and current throughout the data-collection period. All procedures were conducted in accordance with the principles of the Declaration of Helsinki and other applicable international ethical guidelines. Before participation, all participants provided written informed consent after receiving detailed information about the purpose and duration of the study, confidentiality procedures, the voluntary nature of participation, the absence of financial compensation, and their right to withdraw at any time without consequences.

### 2.3. Stimuli

Six news video clips were used as experimental stimuli (see Table 2). The clips ranged from 11 to 98 s, and all stimuli contained limited lower-third on-screen text. All news clips were presented in Spanish. The selected materials represented different audiovisual configurations commonly found in news broadcasts, including segments featuring an on-screen journalist as well as segments without a visible journalist. The clips also varied in topic while all were sourced from the same broadcaster, providing a consistent news-production context across stimuli. This variation was intended to better reflect the diversity of news formats. For the experimental manipulation, each video was prepared in two playback conditions: a normal-speed version (1×) and an accelerated version (2×). The 2× versions were created using iMovie (version 10.4.3), with the “Preserve Pitch” option enabled to maintain the original pitch of the audio. The audio and video tracks were accelerated together rather than processed separately. All stimuli were obtained from the official website of Radio Televisión Española (RTVE) and downloaded in 1080p HD resolution. All selected clips displayed the RTVE logo, ensuring a consistent source identifier across all stimuli. The order of stimulus presentation was randomized using the randomization function implemented in the Gazepoint Analysis software, version 7.2.0 (Gazepoint Research Inc., Vancouver, BC, Canada).

### 2.4. Variables

The independent variable was playback speed, operationalized at two levels: normal-speed playback (1×) and accelerated-speed playback (2×). The audiovisual stimuli remained identical across conditions, with playback speed constituting the only experimental manipulation. Dependent variables were grouped into three domains: self-reported measures, memory-related measures, and eye-tracking measures (see Table 3).

Self-reported measures comprised emotional valence and arousal assessed using the Self-Assessment Manikin (SAM), recommendation intention, and ratings of perceived credibility and perceived comprehension of the news content. The SAM is a validated, nonverbal instrument widely used to assess emotional responses across a variety of contexts. It provides an efficient approach for capturing the affective dimensions of valence and arousal [45,46]. In the present study, valence was assessed using a 9-point SAM scale ranging from 1 (very unpleasant) to 9 (very pleasant), whereas arousal was assessed using a 9-point SAM scale ranging from 1 (very calm) to 9 (very activated). Recommendation intention was assessed using a 0–10 response scale based on the response format used in the Net Promoter Score (NPS) framework [47]. Participants were asked how likely they would be to recommend the playback speed used for the news video to a friend for watching news programs. Perceived credibility was evaluated using a Likert-type scale ranging from 0 (no credibility) to 10 (total credibility), whereas perceived comprehension was evaluated using a Likert-type scale ranging from 0 (no comprehension) to 10 (total comprehension). Participants verbally reported all responses immediately after each video, while the corresponding questions and rating scales were displayed on the screen to facilitate the task.

Objective memory performance was evaluated using a six-item factual recall test, with one question corresponding to each news video clip. The questions required participants to recall specific factual information presented in the corresponding news clips, including information about people, occupations, locations, and events. The questions required brief factual responses, often consisting of a single word. The test was administered immediately after participants had viewed all six stimuli. Memory performance was quantified as the total number of correct responses across the six questions, yielding a score ranging from 0 to 6. An additional open-ended question asked participants which news clip they remembered best; this question was not included in the memory score and was not used in the analyses. The six recall questions, correct responses, and item-level accuracy by playback condition are provided in Supplementary Table S1. Previous research has shown that audiovisual materials facilitate memory encoding because information presented simultaneously through verbal and visual channels tends to improve recall performance [48].

Eye-tracking data were automatically recorded throughout the experimental session using the Gazepoint Analysis system (Gazepoint Research Inc., Vancouver, BC, Canada) and included measures of blinking behavior, fixation activity, pupil diameter, and gaze allocation within a predefined area of interest (AOI). Blink rate was examined as a measure associated with attentional engagement, as spontaneous blinking has been associated with changes in attentional focus and emotional involvement [49]. Higher levels of arousal have also been linked to greater concentration, although the relationship between blink activity and arousal may depend on task context [50]. Moreover, blink patterns have been shown to align with the narrative structure of audiovisual content and to reflect moment-to-moment fluctuations in viewer interest [51,52]. Pupil diameter was recorded during stimulus viewing. Prior studies have demonstrated that auditory and audiovisual stimuli can modulate pupil size [53] and that pupil diameter tends to increase when individuals are exposed to emotionally or visually engaging material [53,54]. These changes have been associated with task demands and mental effort [55,56]. Fixation metrics—specifically fixation rate and fixation duration—were used to characterize eye movement behavior during stimulus viewing. Ocular fixations provide information about gaze allocation and visual information processing, as their temporal characteristics can reflect how viewers process verbal and visual information [57,58]. Finally, eye-tracking data were used to examine gaze allocation within an AOI corresponding to the on-screen captions in both playback-speed conditions. Hereafter, this region is referred to as the lower-third AOI. This AOI encompassed the lower-third captions, which represent a source of text information during news viewing. Although the amount of lower-third text was limited, this region was considered a distinct source of textual information embedded within the news content and was therefore defined as an AOI. Because the 2× videos presented the same content in half the duration of the 1× videos, the percentage of viewing time within the lower-third AOI, rather than absolute viewing time, was used to quantify the relative allocation of gaze to the captions. This approach was informed by previous findings indicating that increased presentation speed reduces rereading and leads to incomplete processing of subtitle content, thereby limiting opportunities for information integration [20]. Accordingly, differences in gaze allocation within the lower-third AOI between normal (1×) and accelerated (2×) playback conditions were examined to determine whether playback speed affects gaze behavior toward lower-third captions in news videos. To ensure consistency across stimuli, the AOI corresponding to the lower-third captions was manually defined over the same lower-third region of the video frame, maintaining constant dimensions across all news video clips. Within the lower-third AOI, fixation-based measures were used to characterize the extent and duration of visual engagement with the captions. This procedure minimized potential confounding associated with differences in AOI size and ensured that the spatial extent of the analyzed lower-third region was consistent across stimuli.

### 2.5. Experimental Design

Experimental sessions were conducted at the Neuro-Com Research Group laboratory at the Universitat Autònoma de Barcelona (116 participants) and at the facilities of Radio Televisión Española in Barcelona (56 participants), Spain. Both sites used the same experimental equipment/setup and comparable low-light viewing conditions. External light sources were blocked at both sites, and the only illumination during the experiments came from the computer and monitor screens. All video stimuli, experimental instructions, and interactions with participants were conducted in Spanish. External noise and disturbances were minimized.

**Figure 1:**
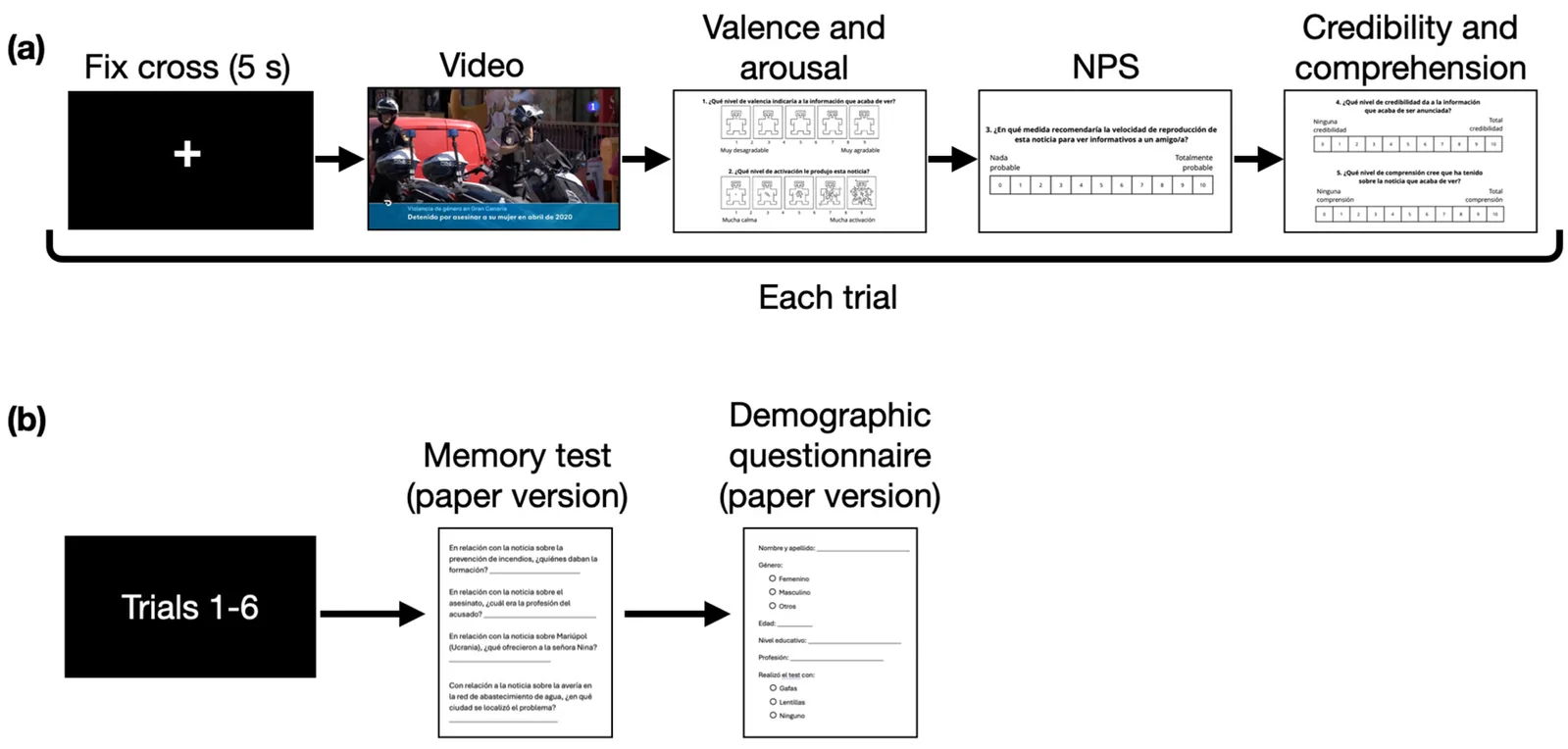
Experimental trial sequence. (**a**) Each trial began with a five-second fixation cross, followed by the presentation of a news video clip under the assigned playback condition—either normal (1×) or accelerated (2×) playback speed. After each video, participants completed three rating screens: the first assessed valence and arousal (SAM), the second assessed recommendation intention using the NPS response format, and the third assessed perceived credibility and perceived comprehension. This procedure was repeated across six trials, with the news video clips presented in randomized order. (**b**) After completing all trials, participants performed a paper-based memory test and subsequently completed a demographic questionnaire.

Each experimental session followed a standardized protocol and was conducted by the same researcher. Upon arrival, participants were seated comfortably and asked to remove potential distractions, such as mobile phones and other sources of distraction. After receiving procedural information and signing the consent form, participants were guided to the experimental station and instructed to maintain a stable posture with minimal head movement. The calibration procedure required participants to visually track a white circle displayed on the screen. Calibration was accepted when the system reported an accuracy score of 4/5 or 5/5 for both eyes. Participants then proceeded with the playback-speed condition assigned to that experimental day. The six news video clips were presented in randomized order. Before each video, a central fixation cross was displayed for five seconds to re-center participants’ gaze and provide a common starting point for each trial. After each clip, participants verbally reported their ratings of emotional valence, arousal, recommendation intention using the NPS response format, perceived credibility, and perceived comprehension of the corresponding news video clip. The researcher recorded these responses on paper forms to avoid disrupting gaze behavior and calibration. Upon completion of all news video clips, participants completed a paper-based memory test and a demographic questionnaire. The duration of each experimental session was approximately 20–25 min. The overall experimental procedure is illustrated in Figure 1.

### 2.6. Apparatus

Data acquisition was performed using a desktop computer (HP ProDesk 600 G5 MT; Intel Core i7 processor, 3.20 GHz, 16 GB RAM). Stimuli were presented on a 27-inch monitor (HP 27fw). Eye-tracking data were recorded using a remote screen-based GP3-HD eye tracker (Gazepoint, Gazepoint Research Inc., Vancouver, BC, Canada). Gaze position was sampled at a frequency of 150 Hz. The GP3-HD eye tracker has previously been shown to be a reliable instrument for psychophysiological research [59]. The distance between the display and the participants’ eyes was approximately 60 cm throughout the experiment. Participants listened to the audio accompanying the news video clips through Sennheiser HD-200 Pro studio headphones. A camera was used to monitor participants during the session. Eye-tracking data were collected using Gazepoint Analysis UX Edition software (v7.2.0), with calibration performed via Gazepoint Control (Gazepoint Research Inc., Vancouver, BC, Canada).

### 2.7. Data Collection and Pre-Processing

Eye-tracking data were recorded throughout stimulus presentation. Stimuli were presented on the primary screen, whereas a second screen was used by the researcher to monitor gaze position, participant video feed, and recording status during the session. Raw gaze and fixation data were exported from Gazepoint Analysis UX Edition in CSV format for subsequent processing. Self-reported responses were initially recorded on paper and subsequently digitized using Google Forms (Google LLC, Mountain View, CA, USA) and Microsoft Excel (Microsoft Corporation, Redmond, WA, USA).

An a priori filtering criterion was applied to the eye-tracking datasets to remove records associated with procedural screens that were not part of the experimental stimuli. Data quality was then evaluated at the video-trial level by reviewing the recorded eye-tracking data for each participant and stimulus. Video trials were excluded when problems with the eye-tracking recording or other technical issues resulted in unusable data for the corresponding trial. The affected video was removed entirely from the dataset for that participant, while the participant remained in the study when sufficient valid trials were available. Participants were excluded entirely when data from four or more of the six video trials were deemed unusable (i.e., ≥60% of the six trials). Participants who met this criterion were excluded from subsequent analyses. Some participants also withdrew voluntarily from the study. As explained above, in total, 26 participants were excluded from the study, with 21 from the 1× condition and 5 from the 2× condition. To assess whether this unequal attrition was associated with systematic differences in the quality of the gaze data retained for analysis, we compared the participant-level proportion of valid gaze samples between conditions. The proportion of valid gaze samples was highly similar in the 1× condition (*M* = 93.96%, *SD* = 4.43) and the 2× condition (*M* = 93.69%, *SD* = 5.34), with no significant between-condition difference (Mann–Whitney *U* = 2596, *p* = 0.864, rank-biserial *r* = 0.017, 95% CI [−0.170, 0.203]). Thus, there was no evidence that the final 1× and 2× samples differed systematically in the quality of the gaze data used for analysis.

Rate-based measures were first calculated separately for each stimulus by normalizing the number of events to stimulus duration and were subsequently averaged across stimuli for each participant. This approach gave equal weight to each stimulus, irrespective of its duration, preventing longer clips from disproportionately influencing participant-level estimates. Eye-tracking measures were calculated using standardized metrics to facilitate comparability across participants and stimuli. Blink-related metrics included spontaneous blink rate (SBR), expressed as blinks per minute, and blink duration in seconds (s). SBR was calculated separately for each stimulus by dividing the total number of blinks by the corresponding stimulus duration and multiplying by 60; the resulting stimulus-level rates were then averaged across stimuli for each participant. Fixation metrics included fixation rate, expressed as fixations per second, and fixation duration in seconds (s). Fixation events were identified using the dispersion-based fixation filter implemented in Gazepoint Analysis UX Edition, using a spatial criterion of approximately 1° of visual angle and a minimum fixation duration of 100 ms. The same fixation-detection procedure was applied to both playback conditions. Fixation rate was calculated separately for each stimulus by dividing the number of fixations by the corresponding stimulus duration and was subsequently averaged across stimuli for each participant. Pupil diameter in millimeters (mm) was used to characterize pupil size during stimulus viewing. Pupil-diameter data were analyzed without baseline correction. For the statistical analyses, pupil-diameter values were averaged across the valid eye-tracking data available for each participant, yielding one mean pupil-diameter value per participant. Additionally, viewing time within predefined AOIs was calculated as the percentage of total stimulus duration during which gaze was directed to each AOI. AOIs were defined using the Gazepoint Analysis UX Edition software (v7.2.0) and corresponded to the lower-third AOI displayed on screen in each stimulus. This region was manually positioned for each stimulus in both playback conditions (1× and 2×) while maintaining a constant size to allow comparison of viewers’ visual attention to the lower-third captions. Fixations longer than 1 s were excluded to reduce the influence of extreme values. This threshold was defined as a preprocessing criterion to reduce the influence of extreme fixation-duration values and was applied identically to both playback conditions. Because long fixations during dynamic viewing may nevertheless represent genuine gaze behavior, the potential influence of this exclusion criterion is acknowledged as a limitation. Data quality was evaluated at the stimulus level, and stimuli associated with frequent gaze loss were removed from subsequent analyses. Details of the excluded video trials by stimulus and playback condition are provided in Supplementary Table S1.

### 2.8. Data Analysis

Statistical analyses were performed using JASP 0.95.3 (University of Amsterdam) with additional analyses conducted using custom MATLAB scripts, version R2025b (MathWorks Inc., Natick, MA, USA). Descriptive statistics were calculated for all variables, including percentages, means, and standard deviations, to summarize the distribution of responses across the two experimental conditions. All inferential analyses were conducted at the participant level. For measures collected repeatedly across the six video stimuli, participant-level aggregate scores were calculated before statistical analysis, such that each participant contributed a single value for each measure. Normality was assessed using the Shapiro–Wilk test, and homogeneity of variances was assessed using Levene’s test. The results of the Shapiro–Wilk and Levene’s tests for each outcome, together with the corresponding statistical test selected, are reported in Supplementary Table S1. To examine differences between playback speed conditions (1× vs. 2×), Student’s independent-samples *t*-tests were used when the normality and homogeneity-of-variance assumptions were met. When normality was met, but homogeneity of variances was not, Welch’s independent-samples *t*-test was used. When the normality assumption was not met, the nonparametric Mann–Whitney U test was used. Cohen’s *d* was reported as the effect size for the independent-samples *t*-test, whereas the rank-biserial correlation was reported for Mann–Whitney U tests. Effect sizes were accompanied by 95% confidence intervals. To account for multiple comparisons, *p* values were adjusted using the Holm procedure across the primary inferential tests, and both unadjusted and Holm-adjusted *p* values are reported. All statistical tests were two-tailed, and the level of statistical significance was set at α = 0.05. For descriptive purposes, recommendation-intention responses were additionally summarized using the conventional NPS classification. The six recommendation-intention ratings were first averaged for each participant, and the resulting participant-level mean was subsequently classified according to the NPS thresholds: scores of 9–10 were classified as promoters, 7–8 as passives, and scores of 0–6 as detractors [47]. The NPS was calculated as the percentage of participants classified as promoters minus the percentage classified as detractors.

## 3. Results

### 3.1. Self-Reported Data

**Figure 2:**
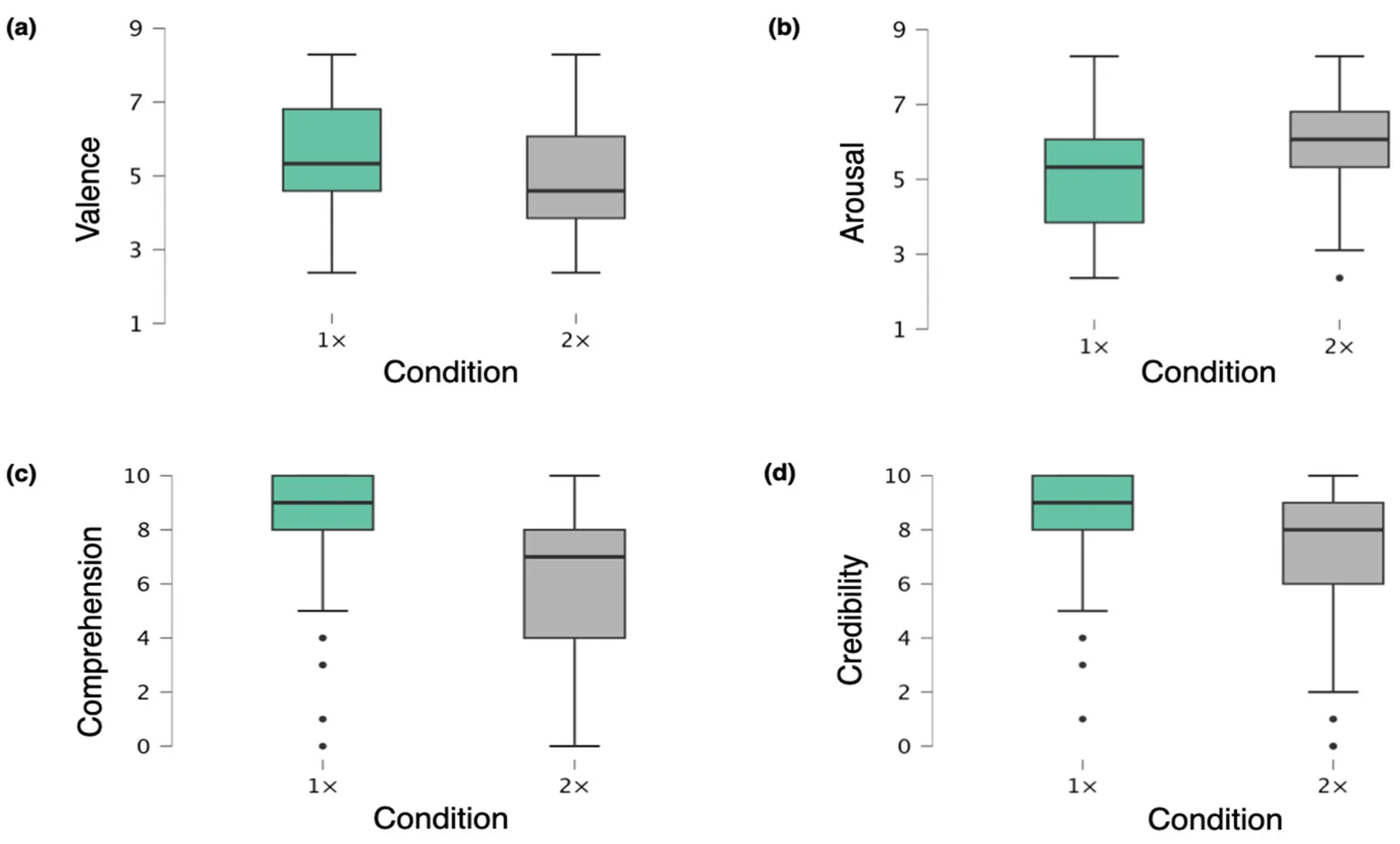
Boxplots of self-reported data for the 1× and 2× playback conditions: (**a**) valence ratings on a 1–9 scale; (**b**) arousal ratings on a 1–9 scale; (**c**) perceived comprehension ratings on a 0–10 scale; and (**d**) credibility ratings on a 0–10 scale. The 1× condition is presented in green, and the 2× condition is presented in gray. Dots indicate individual observations identified as outliers.

Participants reported their evaluations of emotional valence and arousal, as well as perceived comprehension and credibility of the news content (Figure 2), Net Promoter Score, and memory-related measures.

#### 3.1.1. Emotional Valence

Valence ratings spanned the full 1–9 scale in both playback conditions. Mean valence was higher in the 1× condition (*M* = 5.16, *SD* = 1.62) than in the 2× condition (*M* = 4.33, *SD* = 1.50). The Mann–Whitney U test revealed that the difference between the conditions was statistically significant (*U* = 3344.5, *p* = 0.006, Holm-adjusted *p* = 0.028, rank-biserial correlation = −0.267, 95% CI [−0.432, −0.084]).

#### 3.1.2. Arousal

Arousal ratings covered the full 1–9 scale across both playback conditions. Mean arousal was lower in the 1× condition (*M* = 4.63, *SD* = 1.35) than in the 2× condition (*M* = 6.03, *SD* = 1.32). An independent-samples Student’s *t*-test revealed a statistically significant difference between playback conditions (t_(144)_ = −6.291, *p* < 0.001, Holm-adjusted *p* < 0.001, Cohen’s *d* = −1.046, 95% CI [−1.392, −0.697]).

#### 3.1.3. Perceived Comprehension

Comprehension ratings differed across playback conditions, with scores spanning the full 0–10 scale. Participants in the 1× condition reported higher perceived comprehension (*M* = 8.59, *SD* = 1.00) than those in the 2× condition (*M* = 6.10, *SD* = 2.07). The Mann–Whitney U test revealed a significant difference between playback speeds, with a large effect size, indicating lower perceived comprehension in the 2× condition (*U* = 4569.5, *p* < 0.001, Holm-adjusted *p* < 0.001, rank-biserial correlation = −0.731, 95% CI [−0.807, −0.630]).

#### 3.1.4. Credibility

Credibility ratings varied across playback conditions, with responses spanning the full 0–10 range. Participants in the 1× condition assigned higher credibility scores (*M* = 8.74, *SD* = 1.15) than those in the 2× condition (*M* = 7.03, *SD* = 2.10). A Mann–Whitney U test revealed a statistically significant difference between playback conditions (*U* = 4045, *p* < 0.001, Holm-adjusted *p* < 0.001, rank-biserial correlation = −0.532, 95% CI [−0.654, −0.384]).

#### 3.1.5. Recommendation Intention and Net Promoter Score (NPS)

Participant recommendation ratings were evaluated using the Net Promoter Score response format derived from a 0–10 scale. The results revealed substantial differences between playback conditions. The 1× condition yielded a positive NPS (13.64), reflecting a higher proportion of promoters (33.33%) than detractors (19.70%). In contrast, the 2× condition showed a markedly negative NPS (−98.75), characterized by a predominance of detractors (98.75%) and no promoters. For descriptive NPS calculation, the six recommendation ratings were first averaged within each participant, such that each participant contributed a single recommendation score. Consistent with these findings, mean recommendation scores were considerably higher under normal playback (1×; *M* = 8.07, *SD* = 1.63) than under accelerated playback (2×; *M* = 2.14, *SD* = 2.05), indicating a substantial reduction in participants’ willingness to recommend videos presented at higher playback speeds. The Mann–Whitney *U* test confirmed a statistically significant difference in recommendation intention between playback conditions (*U* = 5187.5, *p* < 0.001, Holm-adjusted *p* < 0.001, rank-biserial correlation = −0.965, 95% CI [−0.976, −0.949]).

### 3.2. Memory

Memory performance was assessed using the six-item post-exposure recall test described above. Participants in the 1× playback condition obtained a higher mean number of correct responses (*M* = 2.73, *SD* = 1.13) than those in the 2× playback condition (*M* = 2.05, *SD* = 1.18). A Mann–Whitney U test indicated a statistically significant difference between playback conditions (*U* = 3552.5, *p* < 0.001, Holm-adjusted *p* = 0.002, rank-biserial correlation = −0.346, 95% CI [−0.500, −0.170]).

Table 4 presents the distribution of correct and incorrect responses across playback conditions. Participants in the 1× condition showed a higher proportion of correct responses (45.45%) than those in the 2× condition (34.17%).

### 3.3. Eye-Tracking Data

**Figure 3:**
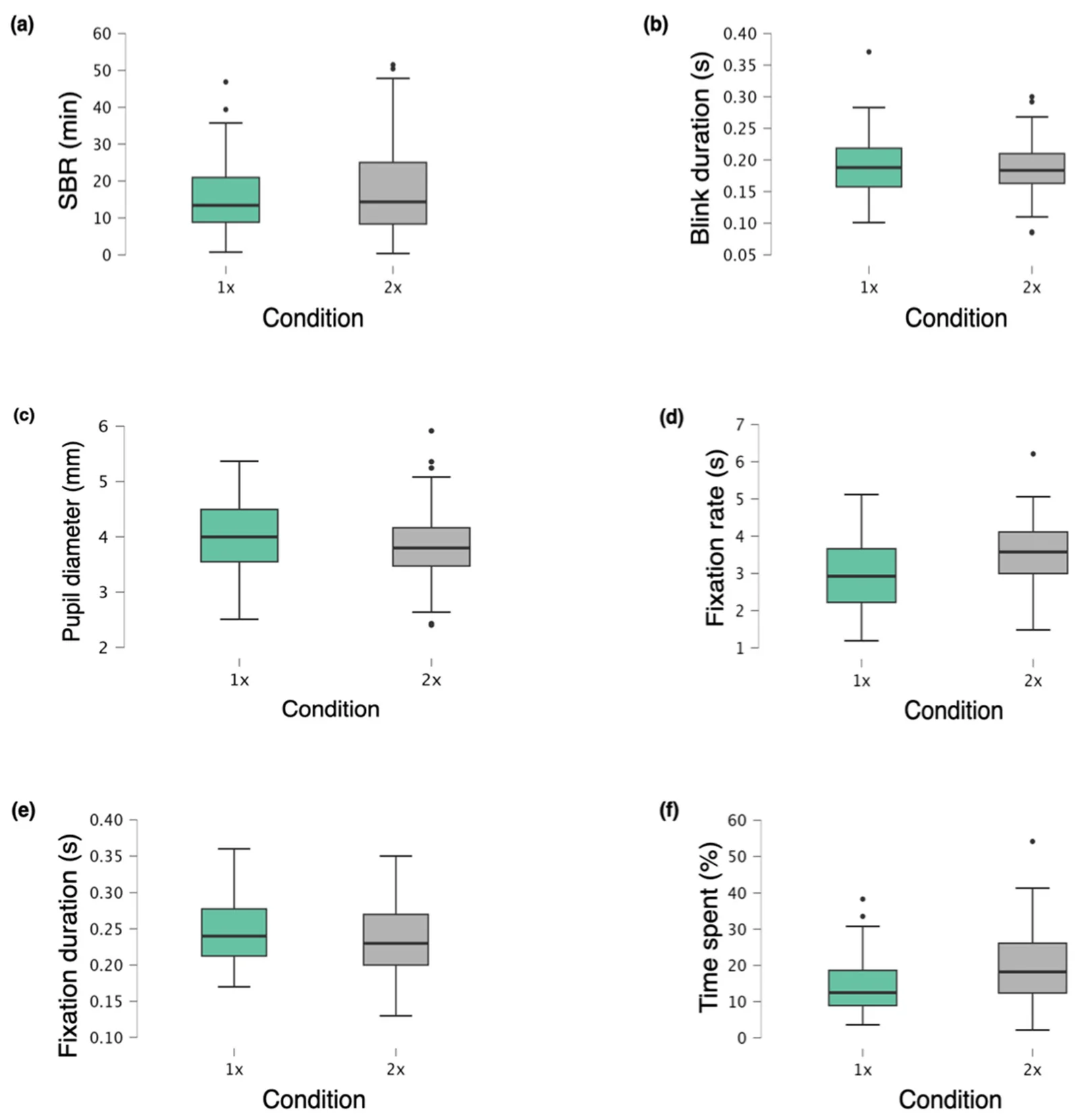
Boxplots showing the distributions of eye-tracking measures across playback conditions. Panels show (**a**) spontaneous blink rate (SBR, blink/min), (**b**) blink duration (s), (**c**) pupil diameter (mm), (**d**) fixation rate (fixations/s), (**e**) fixation duration (s), and (**f**) viewing time within the lower-third AOI (%). Green represents the 1× condition, and gray represents the 2× condition. Dots indicate individual observations identified as outliers.

Eye-tracking measures examined in this section included spontaneous blink rate, blink duration, pupil diameter, fixation metrics (fixation rate and fixation duration), and the percentage of viewing time within the predefined lower-third area of interest (Figure 3).

#### 3.3.1. Blink Behavior

Spontaneous blink rate (SBR) per minute was compared between the two playback conditions. Participants exposed to the accelerated condition (2×) showed slightly higher blink rates (*M* = 17.85 min^−1^, *SD* = 12.03) than those in the normal-speed condition (1×; *M* = 15.20 min^−1^, *SD* = 9.72). The comparison between groups with the Mann–Whitney U test indicated that the difference between playback conditions was not statistically significant (*U* = 2375, *p* = 0.298, Holm-adjusted *p* = 0.597, rank-biserial correlation = 0.100, 95% CI [−0.088, 0.282]).

Blink duration was also compared between the two playback speed conditions. Participants in the normal-speed condition (1×) showed almost identical blink durations (*M* = 0.19 s, *SD* = 0.05) to those in the accelerated condition (2×; *M* = 0.19 s, *SD* = 0.04). The analysis indicated that the observed difference between playback conditions was not statistically significant (*U* = 2759.5, *p* = 0.640, Holm-adjusted *p* = 0.640, rank-biserial correlation = −0.045, 95% CI [−0.230, 0.143]).

#### 3.3.2. Pupil Diameter

Participants in the normal-speed condition (1×) exhibited slightly higher pupil diameter values (*M* = 4.02 mm, *SD* = 0.62) than those in the accelerated condition (2×; *M* = 3.85 mm, *SD* = 0.65). An independent-samples Student’s *t*-test revealed no statistically significant difference between playback conditions (*t*_(144)_ = 1.647, *p* = 0.102, Holm-adjusted *p* = 0.407, Cohen’s *d* = 0.274, 95% CI [−0.054, 0.601]).

#### 3.3.3. Fixations

Fixation rate was compared between playback conditions. Descriptive statistics indicated that participants in the accelerated condition (2×) produced a higher mean fixation rate (*M* = 3.54, *SD* = 0.81) than those in the normal-speed condition (1×; *M* = 2.98, *SD* = 0.96). Welch’s independent-samples *t*-test indicated a statistically significant difference between playback conditions (*t*_(127)_ = −3.794, *p* < 0.001, Holm-adjusted *p* = 0.002, Cohen’s *d* = −0.636, 95% CI [−0.970, −0.300]).

Fixation duration was also examined across playback conditions. Descriptive statistics indicated slightly longer fixation durations in the normal playback condition (1×; *M* = 0.25 s, *SD* = 0.05) than in the accelerated condition (2×; *M* = 0.24 s, *SD* = 0.05). The Mann–Whitney U test showed that the difference between conditions was not statistically significant (*U* = 3013.5, *p* = 0.142, Holm-adjusted *p* = 0.425, rank-biserial correlation = −0.141, 95% CI [−0.320, 0.046]).

#### 3.3.4. Area of Interest (AOI)

The percentage of viewing time within the predefined lower-third AOI was compared across playback conditions. Participants in the accelerated condition (2×) spent a greater percentage of viewing time within the lower-third AOI (*M* = 19.40%, *SD* = 10.38) than those in the normal playback condition (1×; *M* = 14.27%, *SD* = 7.15). The Mann–Whitney U test indicated a statistically significant difference between playback conditions (*U* = 1823, *p* = 0.001, Holm-adjusted *p* = 0.008, rank-biserial correlation 0.309, 95% CI [0.130, 0.469]).

### 3.4. Age as a Covariate

To assess whether the between-group age difference could account for the observed effects, supplementary analyses were conducted with age included as a covariate. Adjustment for age did not alter the statistical significance or direction of any of the primary results (see Supplementary Table S1).

## 4. Discussion

The present findings indicate that playback speed was associated with differences in subjective evaluations, memory performance, and visual attention during news viewing. Compared with normal-speed playback, accelerated playback was consistently associated with less favorable audience responses, including lower valence, reduced perceived comprehension, poorer memory performance, lower credibility evaluations, and weaker recommendation intentions.

A clear contrast emerged between emotional valence and arousal. Participants exposed to the 2× condition reported higher levels of arousal while evaluating the viewing experience less positively than participants in the normal playback condition. This pattern may indicate that accelerated news consumption increases perceived stimulation while simultaneously reducing viewing pleasantness. Subjective emotional responses and physiological measures may not always follow equivalent patterns during audiovisual viewing [50,54]. The differences observed in perceived comprehension and memory performance provide further evidence on the potential disadvantages associated with accelerated news viewing. Participants exposed to normal-speed playback reported higher perceived comprehension and achieved better immediate memory performance than those exposed to accelerated playback. These findings differ partially from previous research conducted with lecture-based or educational videos, in which playback speeds up to 1.25× or 2× were not necessarily associated with lower comprehension or poorer learning outcomes [3,9,10,13]. Studies have even suggested that higher viewing speeds may be perceived as beneficial in educational contexts [15]. In particular, some viewers reported accelerated playback as an efficient strategy that does not negatively affect learning performance [60]. Nevertheless, the present findings indicate that the effects of temporal acceleration may vary depending on the type of audiovisual material being consumed [7,12], particularly in the context of news videos.

Audience evaluations were also reflected in the credibility and recommendation measures. News videos presented at normal speed received higher perceived credibility ratings, whereas the accelerated condition generated markedly lower recommendation scores. The contrast observed in the NPS further indicates substantially lower acceptance of accelerated news viewing. In informative audiovisual contexts, where credibility constitutes an important dimension of audience evaluation, these findings suggest that accelerated playback may negatively affect viewers’ evaluations of news content [2,60,61,62].

The relationship between viewing time and memory performance provides an additional perspective on accelerated audiovisual consumption. Although 2× playback allows the same audiovisual material to be presented in half the time, participants in this condition demonstrated poorer immediate memory performance than those who viewed the news videos at normal speed. Thus, the temporal advantage inherent in accelerated playback was accompanied by lower memory performance. This finding is consistent with previous research suggesting that temporal compression may increase attentional demands while reducing effective information processing under cognitively demanding viewing conditions [21].

The eye-tracking results further indicate that playback speed was associated with differences in visual attention allocation. Participants exposed to accelerated playback produced a significantly higher fixation rate and allocated a greater percentage of their viewing time to the lower-third AOI than participants in the normal-speed condition. However, the higher fixation rate observed in the 2× condition should be interpreted cautiously, as the increased velocity of the visual content may have influenced fixation classification by the dispersion-based detection algorithm. Therefore, this difference should not be interpreted exclusively as evidence of increased attentional allocation. Importantly, this higher percentage reflects a greater relative allocation of the available viewing time to the lower-third AOI, rather than a longer absolute viewing time, as videos in the 2× condition were presented for half the duration. The increased relative allocation of gaze to the lower-third captions under accelerated playback is particularly relevant given previous evidence that increased presentation speed can restrict opportunities for rereading and complete processing of textual information [20]. However, greater relative viewing time within the AOI should not itself be interpreted as evidence of more effective processing or better comprehension. Indeed, participants in the 2× condition simultaneously reported lower perceived comprehension and demonstrated poorer memory performance. By contrast, no statistically significant differences were detected between playback conditions for spontaneous blink rate, blink duration, pupil diameter, or fixation duration. Although previous research has associated faster visual presentation with reduced blink frequency [21], spontaneous blink rate was numerically higher in the 2× condition in the present study. However, this difference, as mentioned, was not statistically significant and should therefore be interpreted cautiously, particularly given the limited sensitivity of the study to detect small effects and the potential variability of rate estimates derived from shorter stimulus durations. Overall, the significant differences in fixation rate and relative allocation of viewing time within the lower-third AOI indicate that temporal acceleration was associated with changes in gaze behavior during news viewing [63].

## 5. Limitations

Several limitations should be considered when interpreting these findings. The experiment compared only two playback-speed conditions, 1× and 2×, and therefore does not establish whether the observed effects would emerge gradually or non-linearly across intermediate playback speeds, such as 1.25× and 1.5×. The study included six news video clips, all obtained from a single broadcaster, RTVE, which may limit the generalizability of the findings to a broader range of news content and sources. The memory assessment also had a limited number of items, with one factual recall question corresponding to each of the six news clips. This limited item count may have increased the influence of individual item difficulty and random response variability and limits the extent to which the test can characterize memory performance across the different news clips. Some participants were also excluded because of eye-tracking data-quality issues, including calibration errors, intermittent signal loss, and technical difficulties during recording. The final groups differed in mean age, with participants in the 2× condition being older on average than those in the 1× condition. Although playback-speed condition was randomized at the experimental-day level and age was not involved in condition assignment, this imbalance represented a potential confounding factor. However, supplementary analyses including age as a covariate showed that adjustment for age did not alter the statistical significance or direction of any of the primary findings. Therefore, although the age imbalance should be considered when interpreting the findings, it does not appear to account for the observed differences between playback-speed conditions. Because assignment occurred at the day level, participants tested on the same day were clustered within the same condition. The present analyses treated participants as independent observations and did not explicitly model potential day-level clustering. Therefore, unmeasured day-specific factors may have contributed to between-condition variability. Another relevant limitation concerns the pupil-diameter analysis, which should be interpreted with caution because individual baseline pupil size was not normalized and the potential effects of increased temporal frequency of luminance changes at 2× speed were not explicitly controlled. Moreover, luminance-related characteristics of the stimuli were not explicitly quantified or incorporated into the pupil-diameter analysis. Another limitation is that subjective ratings were reported verbally to the researcher, which may have introduced demand characteristics or social-desirability bias, particularly in recommendation-intention ratings. Finally, because fixation classification was performed on dynamic video stimuli, differences in stimulus velocity between playback conditions may have influenced the classification of smooth pursuit and fixation events. The higher fixation rate observed at 2× speed should therefore not be interpreted exclusively as reflecting increased visual attention. In addition, fixations longer than 1 s were excluded as extreme values; although this criterion was applied identically across conditions, genuine long fixations may have been removed differentially. All these limitations should be considered when interpreting the findings and their generalizability.

Future research could examine intermediate playback speeds and determine whether the effects observed at 2× emerge progressively as presentation speed increases. It would also be relevant to examine whether similar patterns of subjective evaluation, memory performance, and visual attention emerge across a broader range of news formats, broadcasters, and audiovisual genres.

## 6. Conclusions

This study examined whether playback speed influences subjective responses, memory performance, and visual attention during news viewing. In general, accelerated playback at 2× speed was associated with lower valence, lower perceived comprehension, poorer memory performance, lower perceived credibility, and lower recommendation ratings compared with normal-speed playback.

Significant differences were also observed in visual attention measures. Participants in the 2× condition showed a higher fixation rate and allocated a greater percentage of their viewing time to the lower-third AOI containing the captions than participants in the 1× condition. However, the higher fixation rate should be interpreted cautiously, as increased stimulus velocity may have affected fixation classification. These findings indicate that accelerated playback affects not only viewers’ subjective evaluations and memory performance but also the allocation of visual attention during news viewing.

Taken together, the findings suggest that presenting news videos at 2× speed may entail disadvantages for the viewing and processing of news content. Although accelerated playback allows the same audiovisual material to be viewed in less time, this temporal advantage was accompanied by less favorable audience evaluations. These results highlight the importance of considering playback speed when evaluating how audiences process and respond to audiovisual news content.

## Figures and Tables

**Table 1:** Age characteristics of participants by playback-speed condition and for the total sample.

Characteristic	1×	2×	Total
Number of participants (*n*)	66	80	146
Age, *M* (*SD*)	30.74 (12.42)	37.35 (14.21)	34.36 (13.78)
Female, *n* (%)	41	53	94 (64.4%)
Male, *n* (%)	24	27	51 (34.9%)
Other, *n* (%)	1	0	1 (0.7%)

**Table 2:** Description of the news video clips used as experimental stimuli. Minor deviations from an exact 2:1 duration ratio reflect frame-level rounding during video playback and encoding.

Video	Duration at 1× (s)	Duration at 2× (s)	Content Description
Fire	79.17	39.57	The news clip presented two firefighters visiting the home of an elderly couple to provide fire prevention training and safety guidance.
Murder	54.73	27.30	The news clip presented the case of a former police officer who was under investigation in Spain for the alleged murder of his wife.
Mariupol	97.93	49.03	The news clip presented Mrs. Nina, who was offered an apartment by the Russian government in exchange for obtaining a Russian passport after losing her home in Mariupol during the war in Ukraine.
Water supply	22.60	11.30	The news clip presented a water supply network failure in a Spanish city caused by construction works.
Mar Menor	32.90	16.43	The news clip presented the brown discoloration of the Mar Menor lagoon following torrential rainfall, which retained thousands of cubic meters of water inland and prevented runoff from reaching the lagoon.
Flood	40.93	20.47	The news clip presented a flood that destroyed a water pipeline, leaving residents without potable water and requiring a tanker truck to supply the affected neighborhood.

**Table 3:** Summary of study variables, measurement scales, and assessed dimensions.

Domain	Variable	Scale/Unit	Dimension Assessed
Self-report	Emotional valence	1–9	Affective evaluation
Self-report	Arousal	1–9	Subjective activation
Self-report	Perceived comprehension	0–10	Perceived understanding
Self-report	Credibility	0–10	Perceived credibility
Self-report	Recommendation	0–10	Willingness to recommend
Memory	Recall performance	0–6 correct responses	Subsequent memory
Eye tracking	Spontaneous blink rate	blinks/min	Blink behavior
Eye tracking	Blink duration	s	Blink behavior
Eye tracking	Pupil diameter	mm	Pupil size
Eye tracking	Fixation rate	fixation/time	Fixation behavior
Eye tracking	Fixation duration	s	Fixation behavior
Eye tracking	Lower-third AOI viewing time	%	Relative gaze allocation

**Table 4:** Distribution of correct and incorrect responses in the memory task across playback speed conditions (1× and 2×). Frequencies and percentages are reported.

Condition	Correct, *n* (%)	Incorrect, *n* (%)	Total
1×	180 (45.45%)	216 (54.55%)	396
2×	164 (34.17%)	316 (65.83%)	480

## Data Availability

The original contributions presented in this study are included in the article/Supplementary Materials. Further inquiries can be directed to the corresponding authors.

## Supplementary Materials

The following supporting information can be downloaded at: https://www.mdpi.com/article/10.3390/jemr19050101/s1. Table S1: Complete Cleaned Dataset and Recorded Measures.

## Abbreviations

The following abbreviations are used in this manuscript:

AOI	Area of Interest
CEEAH	Ethics Commission for Research with Animals and Humans
NPS	Net Promoter Score
RTVE	Radio Televisión Española
SAM	Self-Assessment Manikin
SBR	Spontaneous Blink Rate
UAB	Universitat Autònoma de Barcelona
